# A human antibody derived from original SARS-CoV-2 infection effectively neutralizes omicron

**DOI:** 10.1007/s44307-024-00011-1

**Published:** 2024-01-29

**Authors:** Tingting Li, Bingjie Zhou, Haoyu Dong, Dimitri Lavillette, Dianfan Li

**Affiliations:** 1 Center for Excellence in Molecular Cell Science, Shanghai Institute of Biochemistry and Cell Biology, Chinese Academy of Sciences, 320 Yueyang Road, Shanghai, 200030 China; 2 Shanghai Institute of Immunity and Infection, Chinese Academy of Sciences, 320 Yueyang Road, Shanghai, 200030 China; 3 Pasteurien College, Soochow University, Jiangsu, China; 4 Applied Molecular Virology Laboratory, Discovery Biology Department, Institut Pasteur Korea, Gyeonggi-Do, South Korea

**Keywords:** Broad neutralizing antibody, COVID-19, Escape mutants, SARS-CoV-2, Variant of Concern

## Abstract

SARS-CoV-2 (Severe acute respiratory syndrome coronavirus 2) Variants of Concern (VOCs), such as the Omicron sub-variants, present significant challenges in pandemic control due to their capacity to escape antibodies and breach vaccine protections. Discovering antibodies that can tolerate mutations in VOCs and understanding their underlying mechanisms is crucial for developing therapeutics for COVID-19 patients, particularly those for whom other therapies may be unsuitable. Here, we report the neutralization of the Omicron variant by FD20, a broadly active human monoclonal antibody. In contrast to a clinically approved control antibody, FD20 neutralizes Omicron with comparable IC_50_ values to those observed for previously circulating VOCs and the original strain reported in Wuhan. Leveraging structural information, we provide insights into its resilience against mutations in Omicron. The results encourage the prospective development of FD20 as a therapeutic option for COVID-19 caused by current and potentially future VOCs.

## Introduction

The destructive spread of SARS-CoV-2 variants of Concern (VOCs) and their ability to cause breakthrough infections underscore the need for universal therapeutics, including broadly effective neutralizing antibodies. Omicron strains have demonstrated significant capabilities to evade vaccine-elicited antibodies, charactered by numerous mutations in the surface glycoprotein Spike (S), particularly in its receptor-binding domain (RBD) (Planas 2022). Despite their classification as Omicron variants, the BA.4/5 sublineages can even elude serum from BA.1-infected individuals (Tuekprakhon 2022), emphasizing the urgency of developing broadly active vaccines and therapeutics.

An essential step in SARS-CoV-2 infection involves the attachment of the virus to host cells through the molecular engagement between S-RBD and the receptor angiotensin-converting enzyme 2 (ACE2) (Walls 2020, Hoffmann 2020, Shang 2020). S is a heavily glycosylated, trimeric protein (Wrapp 2020, Watanabe et al. 2020). In the “closed” state, the RBD assumes a “down” conformation, concealing the receptor binding motif (RBM) at the inter-subunit interfaces. In the “open” state, at least one RBD assumes an “up” conformation, exposing the RBM for ACE2 binding (Walls 2020, Wrapp 2020, Henderson 2020). Neutralizing antibodies disrupt the RBD-ACE2 interaction through various mechanisms (Barnes 2020), with some directly interfering with ACE2 binding by binding to the RBM (Barnes 2020, Brouwer 2020, Cao 2020, Hansen 2020, Li 2022). Other antibodies, such as COVA1-16 (Liu 2020)and H014 (Lv 2020), bind to non-RBM regions but still prevent RBD-ACE2 interactions through steric hindrance (Li 2022). Less frequently reported mechanisms involve antibodies locking the S trimer in the “closed” state (Barnes 2020; Liu 2021, Schoof 2020), while others, like CR3022 (a neutralizing antibody originally discovered against SARS-CoV (Huo 2020)), FD20 (Li 2021), Ab08 (Meng 2023), and EY6A (Zhou 2020), destruct the S trimer. EY6A shares a similar epitope with CR3022, while FD20 targets a rarely described, highly conserved epitope.

**Fig. 1:**
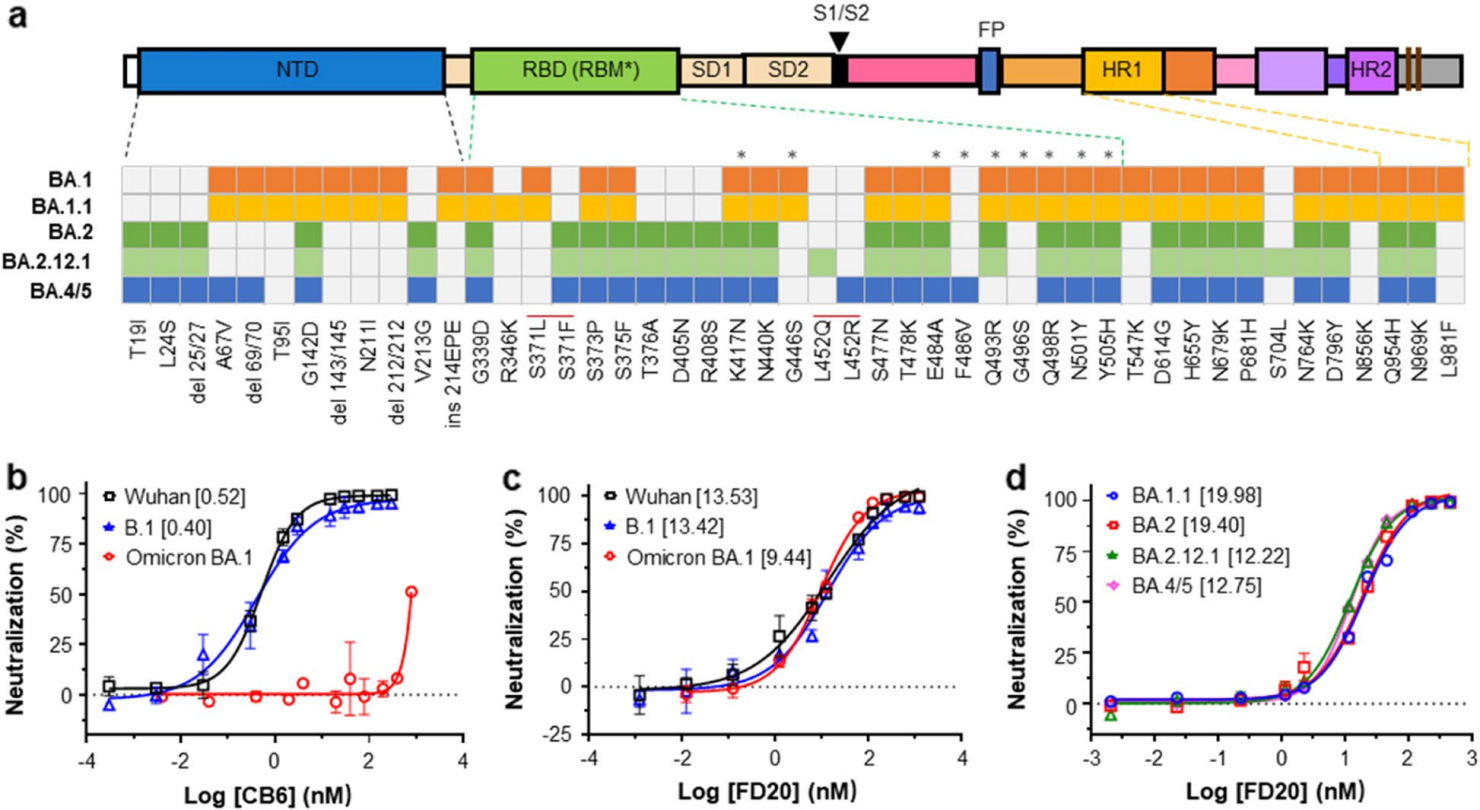
Neutralization activity of FD20 against Omicron. **a** Spike mutations of the Omicron sublineages in this study. Various components are indicated. The occurrence of mutations are color-labeled. NTD, N-terminal domain; RBD, receptor-binding domain; RBM, receptor-binding motif (residues are marked with asterisks). SD1/2, sub-domain 1/2; FP, fusion peptide; HR1/2, heptad repeat region 1/2. **b**,** c** Neutralizing assay of the control antibody CB6 (**b**) and FD20 (**c**) against the original Wuhan SARS-CoV-2 strain (black square), B.1 (D614G) (blue triangle), and Omicron BA.1 (red circle). Retroviral pseudotyped particles were generated in HEK293 cells by transient expression of various viral envelop glycoproteins, the murine leukemia virus core/packaging components, the green fluorescence protein, and the Spike protein of various strains (Wuhan, hCoV-19/China/CAS-B001/2020; B.1, D614G mutant of the Wuhan strain; and Omicron, BA.1 that contained the Spike mutations of the Wuhan strain as indicated in **a**. VeroE6 cells expressing hACE2 were transduced with the pseudotyped particles. Transduction rates were assessed by GFP fluorescence using flow cytometry. IC_50_ values are shown as nM in brackets. Mean and s. d. from three independent experiments are plotted. **d** Neutralizing assays of FD20 against BA.1.1, BA.2, BA.2.12.1, and BA.4/5. IC_50_ values are shown as nM in brackets. Mean and s. d. from three independent experiments are plotted

The RBD is a relatively stand-alone domain with a core region mainly composed of β-strands, and two long loops (the RBM) laying on top of the core region (Lan 2020). Profiling studies suggest that RBM-targeting antibodies are generally more effective than those targeting the core region (Li 2021, Piccoli 2020, Dejnirattisai 2021, Starr 2021). However, the flexible nature of the RBM makes its mutations less structurally constraining than those in the core region (Starr 2020). Consequently, RBM mutations are more prevalent, rendering most RBM-targeting antibodies ineffective (Greaney 2021, Hastie 2021, Nabel 2022, Cao 2022, Chen 2021). For instance, the Omicron variant (BA.1) contains 15 mutations in the RBD (Fig.1a), 10 of which occur in the RBM. The emergence of such variants calls for the development of antibodies that target conserved regions.

Previously, we isolated the abovementioned FD20 from a convalescent COVID-19 patient (Li 2021). FD20 neutralizes a panel of VOCs and well-known escape mutants with similar activity to the original SARS-CoV-2 strain (Li 2021). Remarkably, FD20 targets an “ideal” epitope predicted by saturation mutagenesis in a yeast display system, where mutation frequency correlates negatively with functional (ACE2-binding) and structural constraints (Starr 2020). Further mutagenesis of the epitope using naturally occurring substitutions (Li 2021) reveals that the mutations 1) cause functional or folding defects of S, 2) weaken FD20-binding without compromising neutralizing activity, or 3) are neutral. Finally, FD20 binds SARS-CoV RBD with a similar affinity compared to that of SARS-CoV-2 and displays cross-reactivity against SARS-CoV.

Here, we show that FD20 neutralizes the Omicron sublineages with similar potency to the ones derived from ancestral strain, and provide rationale for its broad activity.

## Materials and Methods

### Expression and purification of neutralizing antibodies

The pDEC plasmids (1.4 mg L^−1^ for light chain, 0.6 mg L^−1^ for heavy chain) carrying the coding sequence of FD20 or CB6 was transfected into HEK293 cells (Expi293) using polyethylenimine at a cell density of 2 × 10^6^ per milliliter. For optimal expression, valproic acid was added to a final concentration of 2 mM. After 48 h of expression, the medium, which contained secreted IgG-FD20 or IgG-CB6, as verified by SDS-PAGE, was collected by centrifugation at 1,000 g for 10 min. The supernatant was filtered through a 0.22-µm membrane, and incubated with Protein A beads for 2 h at 4 °C for batch binding. The beads were then packed into a gravity column and washed with 20 column volume (CV) of PBS buffer. After washing, antibodies were eluted with acidic buffer containing 0.1 M glycine pH 3.0. The eluent was rapidly neutralized by 1 M Tris–HCl pH 8.0 which was pre-added in the collection tubes. NaCl was added to the pooled fractions to a final concentration of 0.15 M. The purified FD20 or CB6 was buffer-exchanged into PBS using a desalting column (Bio-Rad). FD20 was quantified using the theoretical molar extinction coefficient of 109,305 M^−1^ cm^−1^ with absorbance measured using a Nanodrop machine.

### Pseudotyped retroviral particle production, infection, and neutralization assays

Experiments with SARS-CoV-2 pseudovirus were conducted in a P2 level laboratory and with approval from the Institut Pasteur of Shanghai, Chinese Academy of Sciences. The retroviral pseudotyped particles were obtained by co-transfection of HEK293T cells (Cat. CRL-3216, ATCC, tested for free of mycoplasma contamination) using polyethylenimine with the expression vectors that encode the various mutants of SARS-CoV-2 S (Wuhan-Hu-1, GenBank: QHD43419.1), or S from VOCs truncated viral envelope glycoproteins, the murine leukemia virus core/packaging components (MLV Gag-Pol), and a retroviral transfer vector harboring the gene encoding the green fluorescent protein (GFP). Note that the SARS-CoV-2 S variants were all truncated with the removal of the 19 amino-acids at the C-terminus. Pseudotyped particles were harvested 48 h post-transfection by centrifugation. The supernatant fraction was filtered through a 0.45-μm membrane before being used for infection assays.

To evaluate the mAbs neutralization activity against murine leukemia virus (MLV) pseudotyped viruses, VeroE6-hACE2 cells generated previously (Li 2021) (VeroE6 cells: Cat. CRL-1586, ATCC, tested for free of mycoplasma contamination) were seeded at 10,000 cells/well in 48-well plates using DMEM supplemented with 10% FBS, 1% penicillin–streptomycin. After 24 h, serial dilutions of monoclonal antibodies (mAbs) alone, or in combination (1:1 ratio) in 50 µL DMEM were mixed with 100 µL pseudotyped viruses in conditions described in figure legends. After 6 h at 37 ˚C, cell media were changed. The infection was allowed for a further period of 48 h. The cells were then analyzed by analytical fluorescence-activated cell sorting. By comparing to the infectivity of MLV pseudotyped viruses incubated with DMEM medium containing 2% fetal calf serum (FBS) (which was standardized to 100% in the data processing), the neutralization activity of serially diluted mAbs will be calculated.

Mutant SARS-CoV-2 pseudoparticles (pp) were generated by replacing the wild-type S with mutations generated by site-directed mutagenesis. Sequences were verified by DNA sequencing.

## Results

To evaluate FD20’s efficiency in neutralizing the Omicron variant, we packaged the Omicron pseudovirus (BA.1) in HEK293 cells and performed neutralization assays using VeroE6 cells expressing the ACE2 receptor (Yao 2021). An RBM-targeting neutralizing antibody, CB6 (also termed JS016 or LyCoV016 or etesevimab) (Shi 2020), served as a control, and both antibodies were tested in their IgG form.

As shown in Fig. 1b, CB6 displayed potent neutralizing activity against retroviral pseudotypes harboring the original SARS-CoV-2 (termed wildtype, WT) with an IC_50_of 0.52 nM, consistent with reported values in vesicular stomatitis virus (VSV)-derived pseudotypes neutralizing assays (Shi 2020, Cui 2022). While the B.1 mutation (D614G) had no effect on CB6’s neutralizing activity (IC_50_of 0.40 nM), the mutations in BA.1 (Omicron) rendered it ineffective, aligning with findings from lentivirus pseudotypes (Sheward 2022), VSV pseudotypes (Cui 2022, Iketani 2022, Liu 2022, Cameroni 2022)or real virus isolate (Planas 2022, VanBlargan 2022) studies. By contrast, FD20 showed similar potency against the WT, the B.1 strain, and the Omicron strain BA.1 (Fig.1c), maintaining a similar IC_50_ value at approximately 10 nM.

During the study, additional Omicron sublineages, including BA.1.1, BA.2, BA.2.12.1, and BA.4/5, with heightened infectivity emerged. Of particular public concern, BA.4/5, featuring the L452R mutation responsible for antibody escape originally reported in the Delta variant, exhibits high reinfection rate by evading BA.1 serum (Tuekprakhon 2022).

To test FD20’s potency against these sublineages, we constructed pseudoviruses that carry the corresponding S mutations (Fig. 1a). Neutralization assays revealed consistent activities across all sublineages. In detail, although the IC_50_ values for BA.1.1 and BA.2 increased by 1.5-fold compared to the original Wuhan strain, FD20 neutralized BA.4/5 (Fig. 1d) as effective as the original strain (Fig. 1c).

**Fig. 2:**
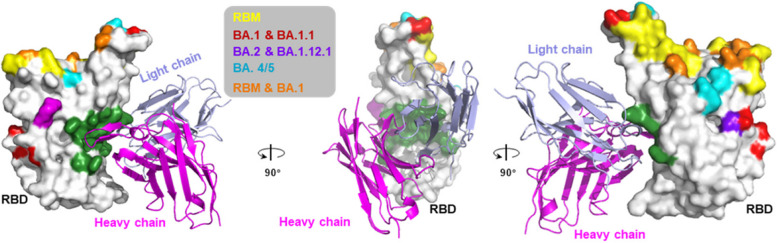
Structure interpretation of FD20’s resistance to the mutations found in Omicron. RBD is shown as surface representations with the receptor-binding motif (RBM) and the mutation sites of the Omicron sublineages highlighted as indicated. The FD20 footprint (green) is distal to the RBM and FD20 neutralizes SARS-CoV-2 by Spike destruction. FD20 (scFv, single-chain variable fragment) is shown as ribbon representations with the heavy chain in magenta and the light chain in light blue. Structures were visulized using PyMOL (version 2.6.0a0, Schrödinger, Inc.)

To rationalize the broad activity of FD20, we mapped the Omicron mutations onto the crystal structure of the RBD-FD20 complex. This analysis provided structural interpretation for the FD20’s resilience to the escape mutations. As shown in Fig. 2, all Omicron RBD mutations were distant from the FD20 epitope, suggesting minimal impact on FD20-RBD interactions. Despite the potential for epitope shape changes due to allosteric effects of the Omicron mutations, FD20's sustained effectiveness against VOCs implies that the VOC mutations do not alter the FD20 epitope, likely because it is situated in the stable core region of RBD. Taken together, the neutralization results and the structural analyses support the assertion that FD20 indeed targets an ‘ideal’ epitope.

## Discussion

The promising results presented herein encourage the future development of FD20 as a broadly active therapeutic antibody against COVID-19 caused by VOCs. Significantly, the omicron BA.1 variant has exhibited resistance to most clinically relevant monoclonal antibodies (mAbs) that are either licensed and authorized for human use or undergoing clinical trials (Planas 2022, Cao 2022, Cui 2022, Sheward 2022, Iketani 2022, Liu 2022, Cameroni 2022, VanBlargan 2022). The mAbs casirivimab (REGN-10933), imdevimab (REGN-10987), etesevimab (Ly-CoV016/CB6), bamlanivimab (Ly-CoV555) and CT-P59 completely lost neutralizing activity against B.1.1.529 virus (Planas 2022, Cao 2022, Cui 2022, Sheward 2022, Iketani 2022, Liu 2022, Cameroni 2022, VanBlargan 2022), with other experienced varying degrees of reduced potency (tixagevimab COV2-2196/AZD8895 and cilgavimab COV2-2130/AZD1061 in combination (Planas 2022, Cao 2022, Cui 2022, Liu 2022, VanBlargan 2022), Brii-196 (amubarvimab) or Brii-198 (romlusevimab) (Cao 2022, Cui 2022, Iketani 2022, Liu 2022)), sotrovimab S309 (Planas 2022, Sheward 2022, Iketani 2022, Liu 2022, VanBlargan 2022), adintrevimab (ADG20) (Planas 2022), and BD-604/DXP-604 (Cao 2022). Notably, a subset of antibodies developed in different laboratories, including S2K146 (ACE2-mimicking antibody), S2X2593, S2H97 (Cameroni 2022), DH1047, S2X259, ADG-2 (Iketani 2022, Liu 2022) BD-744, and S2H97 (Cui 2022) (broadly neutralizing sarbecovirus monoclonal antibodies through recognition of antigenic sites outside the RBM), retained activity against the Omicron BA.1 variant, albeit with varying reduction in potency, warranting further investigation in animal models for future development. However, recent evidence indicated that even mAbs that initially maintained considerable activities against BA.1, such as S309 (sotrovimab), are unfortunately losing their neutralization effectiveness against BA.2 strain (Iketani 2022, McCallum 2022). Furthermore, the current dominant sublineage BA.4/5 can even escape serum from BA.1-infected individuals (Tuekprakhon 2022).

As previously acknowledged, a notable limitation of FD20 is its modest in vivo protection. The implementation of advanced biotechnology, such as structure-based protein engineering optimizations, may hold promise for enhancing its in vivo efficacy. Notably, recent advancements in engineering the Fc domain for selective binding to activating Fcγ receptors have resulted in a fivefold reduction in the effective dose (Yamin 2021). Strategies to improve efficacy include extending antibody half-life through chemical modifications or fusion with albumin-binding partners (Li 2021). Additionally, the engineering of homo or hetero divalent FD20 antibodies presents a viable avenue to bolster in vivo efficacy. The breadth of FD20's spectrum, as previously and currently reported, underscores its significance and calls for heightened research efforts to propel FD20, along with other antibodies targeting this epitope, into advanced stages of drug development to address both current and potential future Variants of Concern (VOCs).

## Data Availability

Data are contained within the article.
